# Quinolylnitrone 23 Protects from Auditory Cell Oxidative Injury and Noise-Induced Hearing Loss

**DOI:** 10.1021/acsptsci.5c00221

**Published:** 2025-08-26

**Authors:** Silvia Murillo-Cuesta, Julio Contreras, Mourad Chioua, Carmen García-Montoya, Lourdes Rodríguez-de la Rosa, Inés Méndez-Grande, Dorota G. Piotrowska, Iwona E. Głowacka, Isabel Varela-Nieto, José Marco-Contelles

**Affiliations:** † Institute for Biomedical Research “Sols-Morreale”, Spanish National Research Council-Autonomous University of Madrid (CSIC-UAM), 28029 Madrid, Spain; ‡ Centre for Biomedical Network Research on Rare Diseases (CIBERER), Institute of Health Carlos III (ISCIII), 28029 Madrid, Spain; § Hospital La Paz Institute for Health Research (IdiPAZ), 28046 Madrid, Spain; ∥ Anatomy and Embryology Department, Faculty of Veterinary, 83137 Universidad Complutense de Madrid, 28040 Madrid, Spain; ⊥ Laboratory of Medicinal Chemistry, Institute of Organic Chemistry (CSIC), Madrid 29006, Spain; # Bioorganic Chemistry Laboratory, Faculty of Pharmacy, Medical University of Lodz, Muszynskiego 1, 90-151 Lodz, Poland

**Keywords:** antioxidants, oxidation-mediated hearing loss, cochlear injury, HEI-OC1 auditory cells, *Nrf2*, *Nlrp3*

## Abstract

Oxidative stress is a key pathogenic mechanism in noise-induced hearing loss, occurring when the production of free radicals in the cochlea overwhelms its antioxidant defenses. Thus, antioxidant molecules, including *N*-acetyl-l-cysteine, acetyl-L-carnitine, resveratrol, HPN-07, and 4-OHPBN nitrones, have been explored as otoprotective agents with limited success. A novel quinolylnitrone derivative QN23 has been shown to suppress oxidative stress in ischemic stroke. In this study, we show that QN23 was not ototoxic and protected from oxidative stress both *in vitro* in the cochlear HEI-OC1 cell line and *in vivo* in mice. QN23 increased HEI-OC1 cell survival after H_2_O_2_-induced oxidative stress, showing better effectiveness than *N*-acetyl-l-cysteine. Systemic administration of QN23 in mice was well-tolerated, and significantly reduced acute auditory threshold shifts 1 day postnoise exposure. The protective effects of QN23 were dose- and time-dependent, with optimal results observed when administered twice daily for 3 days, starting 1 h prior to noise exposure. This protection was associated with the duration of the treatment. QN23 normalized the expression of cochlear genes associated with oxidative stress and inflammation, such as *Nrf2*, *Hmox1*, *Nqo1*, *Nlrp3*, *Tnfa*, *Il1b*, *Dusp1*, and *Kim1*, among others, counteracting immediate noise-induced molecular alterations. These results suggest that QN23 effectively mitigates cochlear oxidative damage and that early intervention can block critical molecular changes induced by noise, thereby preserving hearing.

## Graphical abstract

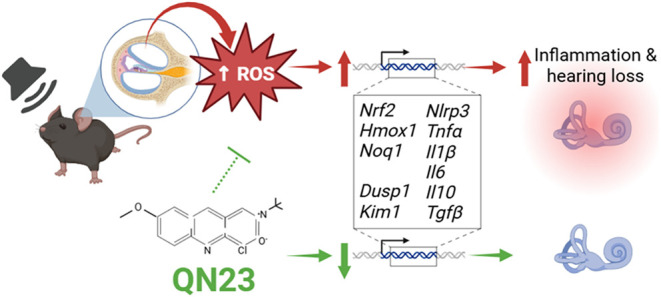

Excessive exposure to noise at work or during recreational activities is a common injury factor for hearing, which contributes to approximately half of the nongenetic hearing loss cases.^1^ Accordingly, noise-induced hearing loss (NIHL) is the second most common form of deafness after presbycusis and constitutes a significant public health concern, given that more than 1 billion young adults are at risk of developing NIHL due to unsafe listening practices.^2^

Excessive noise can injure the cochlea at different levels, loss of ion homeostasis, molecular restructuration, and cellular loss, including the irreversible loss of organ of Corti cells.^3^ The severity of cochlear injury depends on the characteristics of the noise, level, and frequency, as well as on the duration of noise exposure. These factors determine the functional hearing loss consequences that are measured as an increase in the hearing threshold.^4^ Genetic factors influence noise sensitivity; thus, genes involved in antioxidant defense, ion cycling, glutamate excitotoxicity, DNA repair, and other cellular processes have been associated with susceptibility to NIHL in both human and experimental models.^5–7^

The pathological mechanisms underlying noise-induced cochlear injury and hearing loss are diverse depending on the type of exposure (chronic low level, intense and acute, or acoustic trauma) and include mechanical disruption (i.e., hair cell stereocilia), reduced cochlear blood flow, blood-labyrinth barrier alteration, glutamate excitotoxicity or calcium imbalance.^8^ Among them, the excessive production of reactive oxygen and nitrogen species (ROS and RNS) and the subsequent cellular oxidative stress are considered the principal contributors.^9–11^ The molecular basis of oxidative stress in the auditory organ has been studied both *in vitro* and *in vivo* using experimental models. Previous reports with HEI-OC1, a hair cell-like line derived from the immortalized mouse organ of Corti, have shown the accumulation of ROS and oxidative stress-associated injury after exposure to ototoxic agents such as acetaminophen or cisplatin.^12,13^ Similarly, initial studies in animal models established that exposure to intense noise induces the rapid overproduction of ROS,^14^ which can persist even days after noise exposure has ceased.^15^

Under physiological circumstances, ROS generated by mitochondrial activity are scavenged by endogenous antioxidants and antioxidative enzymes (i.e., glutathione, thioredoxins, and glutaredoxins) and maintained at low levels, which are crucial for cellular functioning. When the overproduction of free radicals overwhelms the cochlear antioxidant defenses, accumulated ROS can alter lipids, proteins, and DNA, among other cellular constituents, ultimately leading to the activation of apoptotic signaling pathways.^16^ Vasoactive products generated after lipid peroxidation reduce cochlear blood flow and contribute to ischemia, hypoxia, and further ROS production.^17^

Accumulated ROS induce the expression of genes involved in the early cellular response to oxidative stress through the activation of the nuclear factor erythroid 2-related factor 2 (NRF2).^18^ NRF2 translocates to the nucleus after noise exposure and increases the expression of downstream antioxidant and cytoprotective enzymes heme oxygenase-1 (HO-1) and NAD­(P)H quinone dehydrogenase 1 (NQO1).^19,20^ HO-1 participates in the degradation of heme into biliverdin, iron ion, and carbon monoxide, which have cytoprotective effects by reducing oxidative stress. Similarly, NQO1 contributes to the detoxification of quinones, preventing their participation in redox cycling and subsequent production of more ROS. Thus, pharmacological NRF2 activation can be effective for NIHL prevention.^19^ ROS can also activate the NFKB and the MAPK signaling pathways in cochlear resident macrophages,^21^ leading to the upregulation of inflammatory response genes (*Tnfa*, *Il1b*, and *Il6*) and the production of proinflammatory cytokines, chemokines, and adhesion molecules, which recruit and activate other immune cells that prolong the inflammation, oxidative stress, and consequent tissue injury.^22–24^

Due to the key role of oxidative stress in NIHL, a number of pharmacological approaches have been developed for its prevention or treatment based on antioxidant drugs. Free radical scavengers like ebselen or glutathione replenishers like *N*-acetyl-l-cysteine (NAC) and acetyl-l-carnitine (ALCAR) have been evaluated in NIHL animal models but also in human clinical studies (reviewed in ref 11). The administration of drugs to the cochlea presents an additional challenge due to its complex anatomy and difficult access, which have limited the development of specific medications for the prevention or treatment of hearing loss.

Nitrones are molecular entities characterized by an N-oxide of an imine in their chemical structure, with a general chemical formula X-CH=NO-Y, and are known for their ability to trap free radicals in chemical and biochemical systems. In recent years, certain nitrones, such as phenyl-*N*-*tert*-butylnitrone (PBN), disufenton sodium (NXY-059), and 4-hydroxy PBN (4-OHPBN), have demonstrated biological activity in experimental animal models of diseases where oxidative stress plays a key role, such as stroke, cancer, Parkinson’s disease, and Alzheimer’s disease.^25,26^ Evidence in NIHL is less compelling; thus, Rao and Fetcher reported that PBN was unable to reduce noise-induced injury but was effective in preventing the potentiation by toxins of noise injury.^27^ Choi et al. reported that 4-OHPBN, a major metabolite of PBN, significantly decreased permanent NIHL in chinchillas, with enhanced therapeutic effects when combined with NAC and ALCAR.^28^ Finally, a preclinical study by Erwen and colleagues showed that 2,4-disulfophenyl-*N*-*tert*-butylnitrone (HPN-07) preserved better the structural and functional integrity of the cochlea after noise exposure in chinchillas.^29^

The development of new nitrone derivatives with improved pharmacological properties, higher antioxidant activity, and additional biological effects (e.g., anti-inflammatory activity) is a promising strategy for NIHL drug discovery.^26^ Quinolylnitrones are small molecules synthesized from quinoline carbaldehyde and *N*-alkyl hydroxylamine. Quinolylnitrone derivatives have shown antioxidant and neuroprotective effects during oxygen-glucose deprivation in primary neuronal cultures, that is, (*Z*)-*N*-benzyl-1-(2-chloro-6-methylquinolin-3-yl)­methanimine oxide (RP19) induced significant neuroprotection in experimental ischemia in neuronal cells and against ischemic injury following brain ischemia in treated animals.^30^ More recently, (*Z*)-*N*-*tert*-butyl-1-(2-chloro-6-methoxyquinolin-3-yl)­methanimine oxide (QN23, Table 1) showed a strong antioxidant profile against hydroxyl radicals and provided greater protection and tolerability than RP19 in primary neuronal cultures subjected to oxygen and glucose deprivation, as well as higher prevention of stroke consequences in animal models.^31–34^ Since ischemic stroke and noise-induced cochlear injury share oxidative stress as a central pathogenic mechanism, we hypothesized that quinolylnitrones with therapeutic potential on brain ischemia could also be promising candidates for NIHL treatment.^35^

**Table 1:**
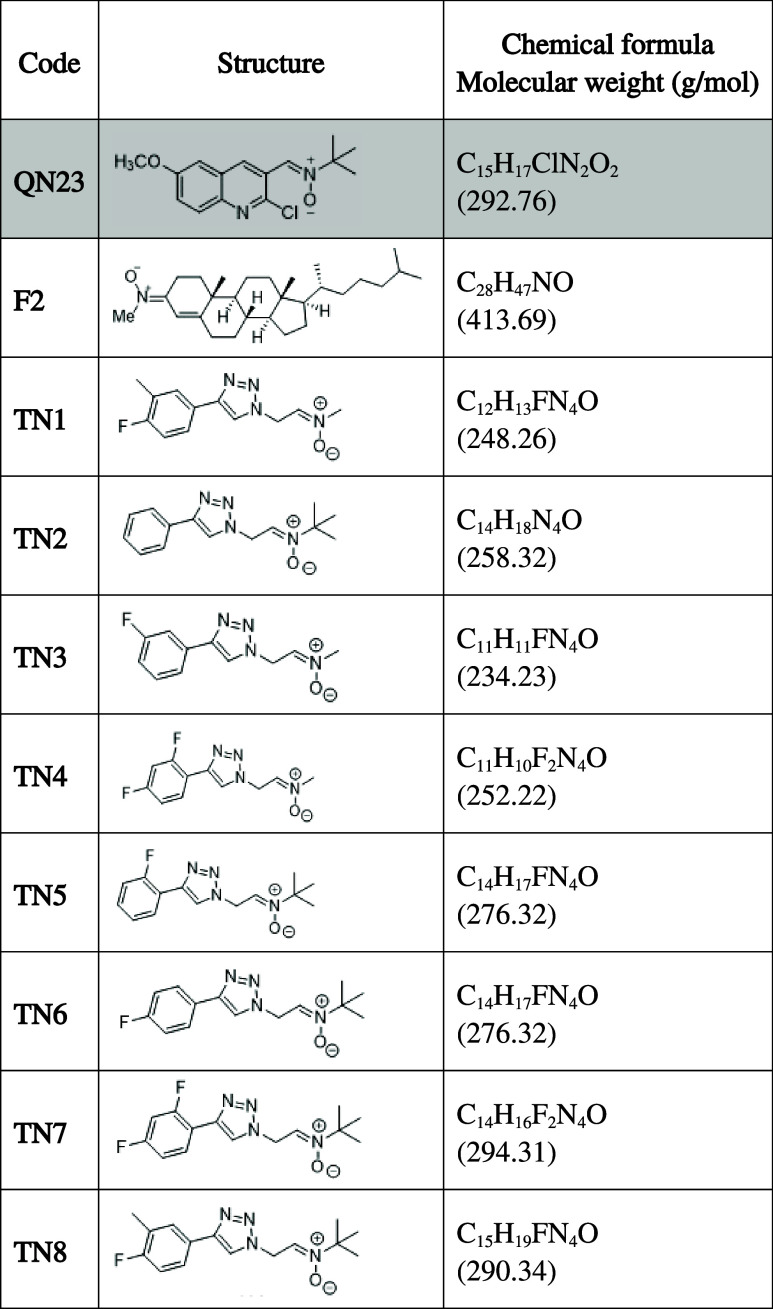
Chemical Structure of Nitrones QN23, F2, and TN1 – TN8

## Results and Discussion

In this study, we evaluated the safety and efficacy of QN23 in preventing oxidative-stress-mediated hearing injury in HEI-OC1 cells exposed to hydrogen peroxide (H_2_O_2_) and in mice exposed to noise.

### Effect of QN23 on HEI-OC1 Cell Viability and Protection against Oxidative Stress

As a preliminary step to determine the otoprotective effects of QN23 *in vivo*, we evaluated its potential toxicity on HEI-OC1 cell viability in the absence of any toxic insult by using the XTT assay. *In vitro* experiments were conducted according to the scheme shown in Figure 1A. Briefly, cells were cultured for 3 days and then subjected to 24 h of serum deprivation. QN23 was added to the culture for 2 h, and then cell viability was assessed by the XTT assay as described in the Methods section. Similar results were observed in all the absorbance reading times, and the 6 h post-XTT addition time is shown.

**Figure 1:**
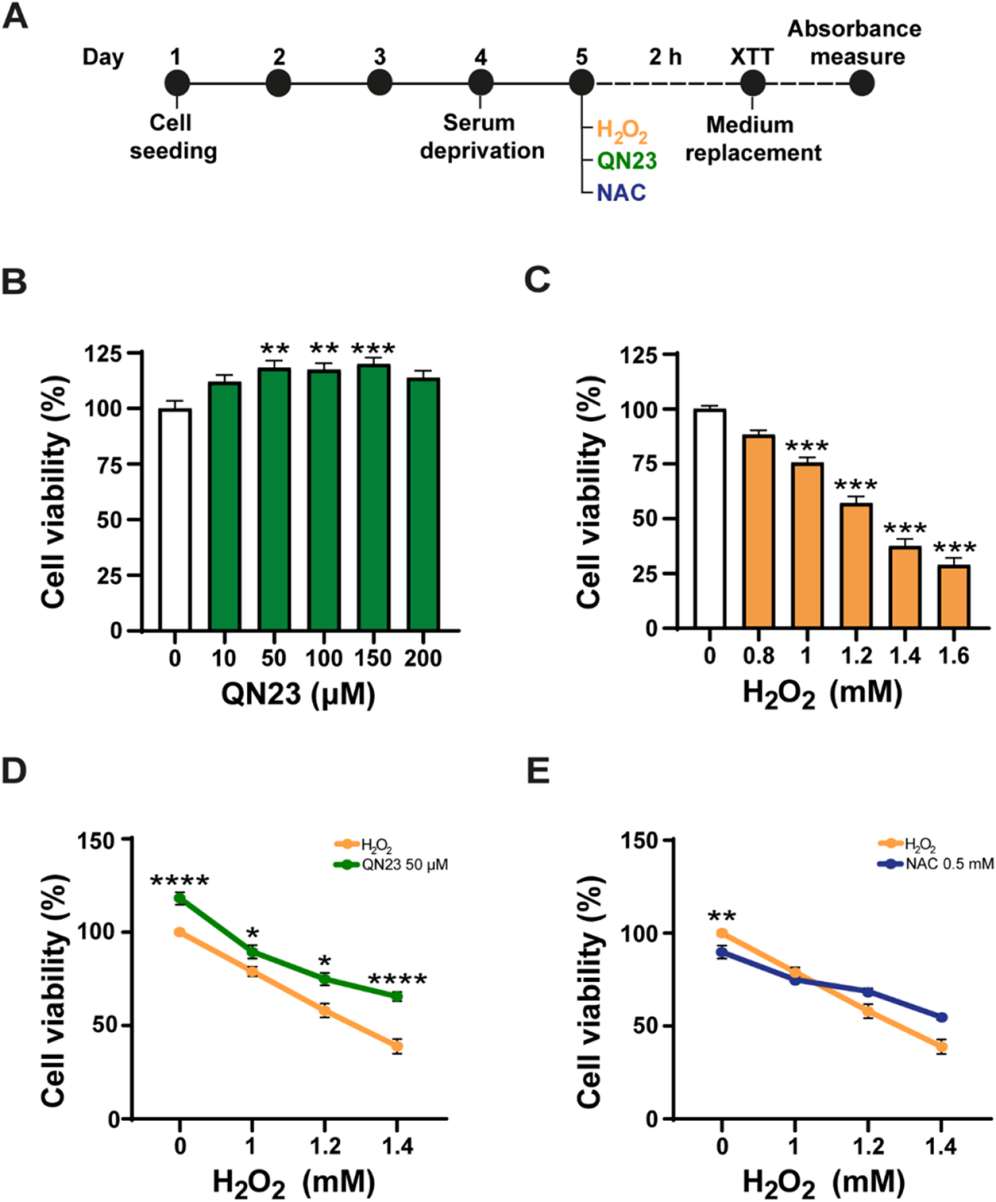
QN23 protects HEI-OC1 auditory cells from oxidative stress. (A) Schematic overview of the experimental design. Cells were cultured for 3 days and then subjected to 24 h of serum deprivation. Cells were exposed to H_2_O_2_ (orange) and nontreated or cotreated with QN23 (green) or NAC (blue) for 2 h. Cell viability was measured using the XTT assay; the 6 h reading time is shown in B-E. Cell-free wells were used as mock controls, and the average optical density measured in untreated cells was considered as 100% viability. (B) Study of the potential toxicity of QN23 (10, 50, 100, 150, and 200 μM) on HEI-OC1 cell viability. (C) Ototoxic effect of H_2_O_2_ (0.8, 1, 1.2, 1.4, and 1.6 mM) on HEI-OC1 cell viability. (D, E) Protective effect of QN23 (D) and NAC (E) against H_2_O_2_-induced oxidative stress. Cells were nonchallenged or challenged with H_2_O_2_ (orange) and untreated or treated with QN23 (50 μM, green) or with NAC (0.5 mM, blue). Results are representative of 2–6 independent experiments performed in septuplicate in independent wells/experiment. All data are presented as mean ± SEM. Statistical significance was estimated using Kruskal–Wallis and Wilcoxon rank-sum nonparametric tests followed by Dunn′s multiple comparison test (*p < 0.05; **p < 0.01; ***p < 0.001).

No substantial ototoxic effects were observed for QN23 at any of the tested concentrations (10–200 μM) or XTT reading time points (4, 6, or 23 h) (Figure 1B). The absence of ototoxic effects across this broad concentration range pointed to QN23 for subsequent otoprotective assay.

After confirming the lack of toxic effects of QN23 within a wide concentration range, we studied its otoprotective properties. HEI-OC1 cells were treated with H_2_O_2_ to simulate cochlear oxidative stress resulting from noise exposure. H_2_O_2_ reacts with Fe^2+^ ions, generating OH radicals, and is widely used to induce oxidative stress in cellular models and to test the efficacy of new antioxidant molecules. First, we evaluated cell viability after challenge with H_2_O_2_ at different concentrations (0.8, 1, 1.2, 1.4, and 1.6 mM) by using the XTT assay (Figure 1C). A significant dose-dependent decrease in cell viability was observed with all of the concentrations tested, as reported.^36^

Next, we assessed the effect of QN23 on H_2_O_2_-challenged cells (Figure 1D). Simultaneous administration of QN23 with the stressor H_2_O_2_ improved cell viability depending on both QN23 and H_2_O_2_ concentrations. Specifically, 1, 1.2, and 1.4 mM H_2_O_2_ challenge caused significant cell death, rendering a cell viability of 79, 58.1, and 38.9%, respectively, whereas upon QN23 treatment, there was a statistically significant dose-dependent increase in cell viability (Figure 1D). Treatment with 50 μM QN23 increased cell viability up to 89.5, 74.9, and 65.6% in the presence of 1, 1.2, and 1.4 mM H_2_O_2_, respectively. The addition of 0.5 mM NAC, a reference antioxidant molecule, only increases cell viability (compared to untreated cells) at the highest H_2_O_2_ concentration tested, with 68.6 and 54.8% in 1.2 and 1.4 mM H_2_O_2_-challenged cells, respectively, but, in contrast to QN23, no significant differences were found (Figure 1E).

In SH-SY5Y human neuroblastoma cells^33,37^ and primary neuronal cultures from rat cerebral cortex,^38^ QN23 was not cytotoxic on a wide concentration range (0.001–100 μM), showing high potency against oxidative stress and significant neuroprotective effects, similar to those reported with NAC. However, QN23 was neurotoxic at concentrations >100 μM^33,39^ on these cell types, whereas no decrease in cell viability was observed in HEI-OC1 cells up to 200 μM. The protective capacity and low toxicity shown at low concentrations, 50 μM, pointed to QN23 as a good candidate for the treatment of oxidation-mediated hearing loss.

In addition to QN23, several other derivatives that included cholesteronitrone F2 ^39^ and triazole-derived nitrones TN1–TN8 ^40^ (Table 1) were also screened for their cytotoxicity and efficacy against H_2_O_2_ challenge in HEI-OC1. Similarly, cytotoxicity of these nitrones was tested in a range of concentrations (10–40 μM), and the resulting cell viability was compared with that of untreated cells. A statistically significant decrease in cell viability was observed when cholesterol nitrone F2 was added at 30 μM; therefore, it was not further tested (Table 2). On the contrary, the triazole-derived nitrones did not decrease cell viability within the tested concentration range or even at higher concentrations (100–150 μM, data not shown) and therefore were selected for efficacy assays against challenge with 1.2 mM H_2_O_2_. Results from efficacy studies were variable: no statistically significant changes in cell viability were observed with TN2, TN4, TN5, and TN7, whereas TN3 and especially TN6 induced a significant viability increase (Table 2). Indeed, quinolylnitrone QN23 was selected for *in vivo* studies as the best in the above-mentioned battery of nitrones although further work is expected to explore TN3 and TN6 therapeutic potential.

**Table 2:** Cytotoxicity and Efficacy Studies of Nitrones QN23, F2, and TN1–TN8^a^

Nitrone		Toxicity (nitrone)			Efficacy (nitrone + 1.2 mM H_2_O_2_)		
QN23	dose (μM)	0	50	100	0	50	100
	viability (%)	100 ± 3.4	111.9 ± 3.2	117.3 ± 3**	44.5 ± 1.8	59 ± 2.4**	74.9 ± 3.3***
F2	dose (μM)	0	10	30	-	-	-
	viability (%)	100 ± 5.2	88.3 ± 5.8	50.2 ± 3.5***	ND	ND	ND
TN1	dose (μM)	0	10	30	0	1	10
	viability (%)	100 ± 9.8	117.8 ± 2.6	129.5 ± 20.9	31.4 ± 1.6	39.4 ± 0.6	37.8 ± 3
TN2	dose (μM)	0	10	40	0	1	10
	viability (%)	100 ± 5.5	112.4 ± 2.9	118.3 ± 2.2*	55.1 ± 3.8	65.2 ± 2.9	61.9 ± 4.1
TN3	dose (μM)	0	10	30	0	1	10
	viability (%)	100 ± 9.1	ND	124.9 ± 12.7	31.4 ± 1.6	41.5 ± 1.7*	30.6 ± 1.4
TN4	dose (μM)	0	10	30	0	1	10
	viability (%)	100 ± 5.2	100.1 ± 1.2	103.1 ± 1.2	31.4 ± 1.6	38.9 ± 2.9	36.9 ± 2.6
TN5	dose (μM)	0	10	30	0	1	10
	viability (%)	100 ± 5.2	101.6 ± 0.8	102.9 ± 1.9	31.4 ± 1.6	27.2 ± 1.5	27.1 ± 0.8
TN6	dose (μM)	0	10	30	0	20	40
	viability (%)	100 ± 5.2	99.6 ± 0.9	95.9 ± 1.7	29.4 ± 5.1	52.4 ± 3.7*	51.8 ± 4.6*
TN7	dose (μM)	0	20	40	0	1	10
	viability (%)	100 ± 5.2	98.2 ± 1.2	108.6 ± 15.4	31.4 ± 1.6	31.0 ± 1.7	33.3 ± 1.5
TN8	dose (μM)	0	10	30	0	1	10
	viability (%)	100 ± 5.2	98.7 ± 2.7	98.2 ± 1.6	31.5 ± 1.6	22.6 ± 1.8*	24.4 ± 1.7*

a HEI-OC1 cells were cultured for 3 days and then serum-deprived for 24 h. For toxicity studies, cells were treated with each nitrone at the indicated doses (μM). For efficacy studies, cells were co-exposed to 1.2 mM H_2_O_2_ and nitrone at the indicated doses (μM) for 2 h. Cell viability was measured using the XTT assay in at least four independent wells/data points. Cell-free wells were used as mock controls, and the average optical density measured in untreated cells was considered as 100% viability. All data are presented as mean ± SEM. Statistical significance was calculated using the nonparametric Kruskal–Wallis test (*p < 0.05; **p < 0.005). ND, not determined.

### Protective Effect of QN23 against Noise Injury in Mice

Earlier studies on the protective effect of QN23 in animal models of ischemic stroke demonstrated significant neuroprotection when administered systemically at 1.5–2 mg/kg.^32,34^ Therefore, we decided to evaluate the protective effect of QN23 at 2 mg/kg in a mouse model of NIHL. Since QN23 is poorly soluble at high concentrations, we used a vehicle composed of ethanol, poly­(ethylene glycol) 400, and saline (1:200:60) by vol, as reported.^34^ To ensure the presence of nitrone and therefore the antioxidant effect during the critical first hours following noise exposure, either nitrone vehicle or nitrone QN23 was administered 1 h before noise and then every 12 h for 3 consecutive days intraperitoneally. Preliminary experiments indicated that a single daily administration for 2 consecutive days had no effect (not shown).

Mice were exposed to a violet swept sine noise (10 s linear frequency sweep from 2 to 20 kHz)^4,41^ at 105 dB SPL during 30 min. Hearing thresholds were determined by recording auditory brainstem responses (ABR) before and 1, 14, and 28 days after noise exposure (Figure 2A). These experimental times inform of the baseline mouse hearing threshold (before noise exposure) and of the acute, temporary, and permanent threshold shifts induced by noise exposure.^3^

**Figure 2:**
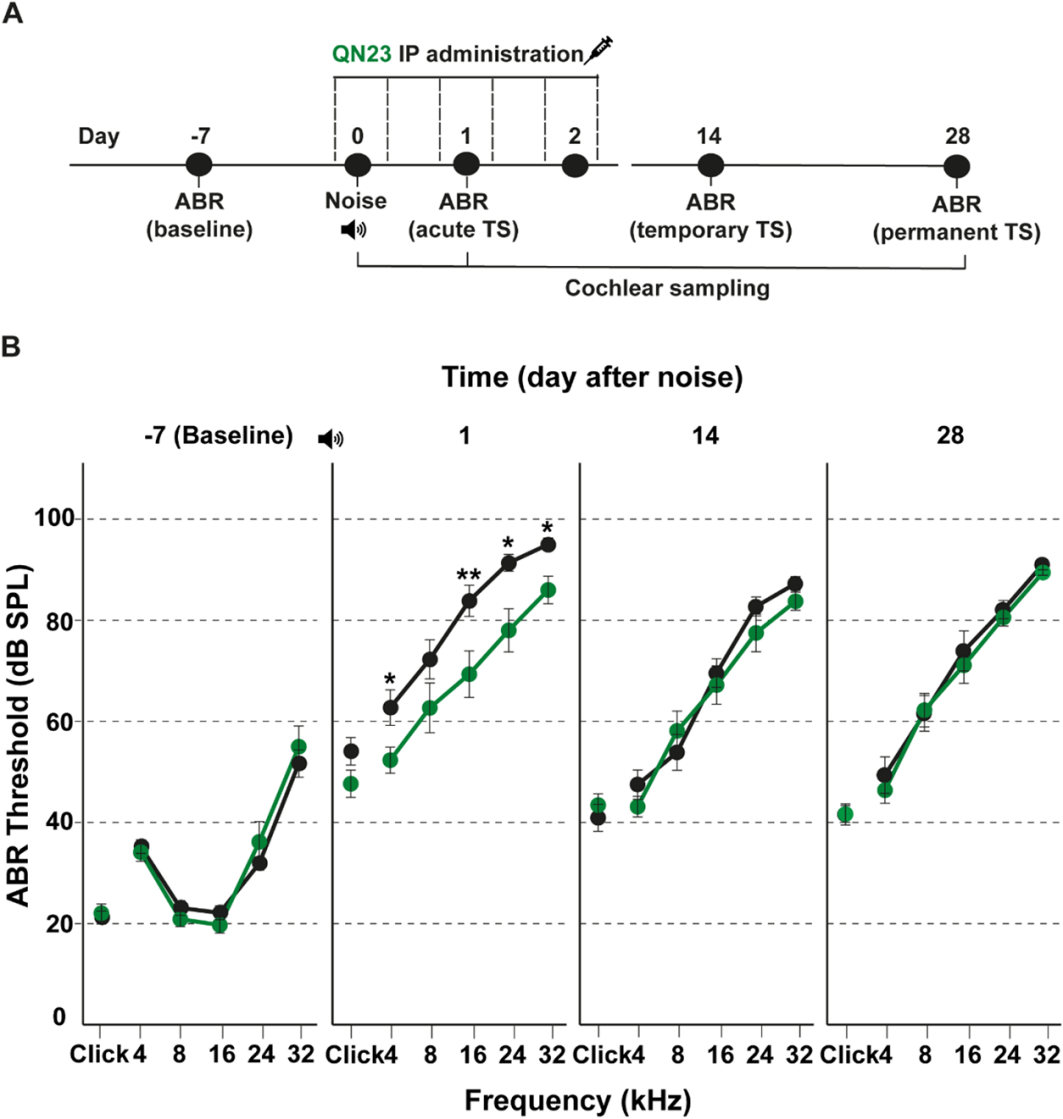
QN23 protects against noise exposure. (A) Schematic overview of the experimental design. Briefly, 2-month-old male C57BL/6JCrl mice were exposed to noise for 30 min to induce NIHL and were either treated with QN23 or left untreated (vehicle). (B) Evolution of auditory thresholds in mice treated with QN23. The protective effect of QN23 on hearing was evaluated by ABR. Baseline thresholds were within the normal-hearing range and increased after noise exposure. Mice treated with QN23 exhibited statistically significantly lower threshold shifts than control animals injected with the vehicle 1 day after exposure. No differences with vehicle-injected mice were observed at the later time points measured after treatment ceased. Data from three independent experiments are shown with a total of 46 mice tested (*n* = 23 QN23-treated and *n* = 23 vehicle-injected). All data are presented as mean ± SEM. Statistical significance was calculated using the nonparametric Kruskal–Wallis test (*p < 0.05; **p < 0.01).

Baseline recordings showed the typical audiogram profile for normal-hearing young C57BL6/J mice (Figure 2B). No differences were observed between the QN23-treated and vehicle-injected groups at this time. However, 1 day after noise exposure, hearing thresholds in the vehicle mouse group increased by over 40 dB in response to both click and tone-burst stimuli, whereas threshold shifts in QN23-treated mice showed less increase (20 dB). Statistically significant differences in ABR thresholds in response to pure tones were observed between the vehicle and QN23 groups, suggesting that QN23 treatment provides all-frequency protection against early cochlear injury and acute threshold shifts following noise exposure. QN23 treatment was ended on the third day after noise exposure, and no further hearing loss recovery was observed thereafter (Figure 2B).

Previous studies with other nitrone derivatives, such as PBN, OH-4-PBN, and HPN-07, showed that the tested nitrones exhibited limited protection by themselves against noise-induced injury, including loud noise, blast noise, and acoustic trauma in different rodent species.^28,29,42,43^ Still, these previous pieces of evidence supported the hypothesis that early intervention with improved nitrone derivatives after noise exposure would efficiently reduce cochlear oxidative stress and preserve the homeostatic and functional integrity of the cochlea. Moreover, the combination of nitrones with other antioxidants, such as NAC, increased the effectiveness of the treatment and reduced the required dose of each drug, thereby minimizing adverse effects.^28,42,43^

The *in vivo* experimental design presented here took into account previous studies with nitrones.^28,29,42,43^ Lu et al.^45^ reported that the average plasma concentrations of HPN-07 and the associated antioxidant status peaked 30 min postinjection and then declined to near baseline levels at 4 h postadministration.^43^ Thus, QN23 intraperitoneal injections in our study were initiated 1 h before noise exposure to ensure nitrone availability in the cochlea and were continued twice daily for 2 days. The QN23 dosage selected had been previously shown to exert a protective effect on experimental models of ischemic stroke.^34^ Specific pharmacokinetic studies on QN23 biodistribution in the cochlea, which are beyond the scope of this paper, should be conducted to determine cochlear nitrone levels. Further work would also be required to study the feasibility of local administration to the inner ear, which could be a promising option, as shown for other small molecules with different targets.^44,45^

The protective effect obtained by QN23 is comparable to that obtained using other nitrones but at much higher doses. For instance, Ewert et al. reported 10 dB lower ABR thresholds 1 day after noise exposure in animals treated with 300 mg/kg HPN-07 compared to the control group.^29^ Choi et al. observed a dose-dependent otoprotective response for 4-OHPBN, with maximum protection at 75 mg/kg.^28^ The combination of antioxidants with different mechanisms of action is an effective strategy to reduce these doses. Thus, the enhanced protective effect of nitrones combined with NAC has been reported to increase the level of protection of these high nitrone doses.^42,43^

To assess the cytoarchitecture of the hearing receptor, cochlear samples were obtained 28 days after noise exposure in both experimental groups to evaluate the gross morphology. Analysis of hematoxylin–eosin-stained paraffin sections evidenced typical noise-induced cochlear morphological alterations, mainly loss of hair cells, collapse of the tunnel of Corti, and loss of neural fibers, in the basal region, as described previously by us in C57BL/6JCrl mice exposed to VSS noise.^46^ Individual differences were found in noise-induced injuries in both experimental groups, vehicle-injected and QN23-treated mice, although mice treated with QN23 showed less extension of the lesions (Figure 3).

**Figure 3:**
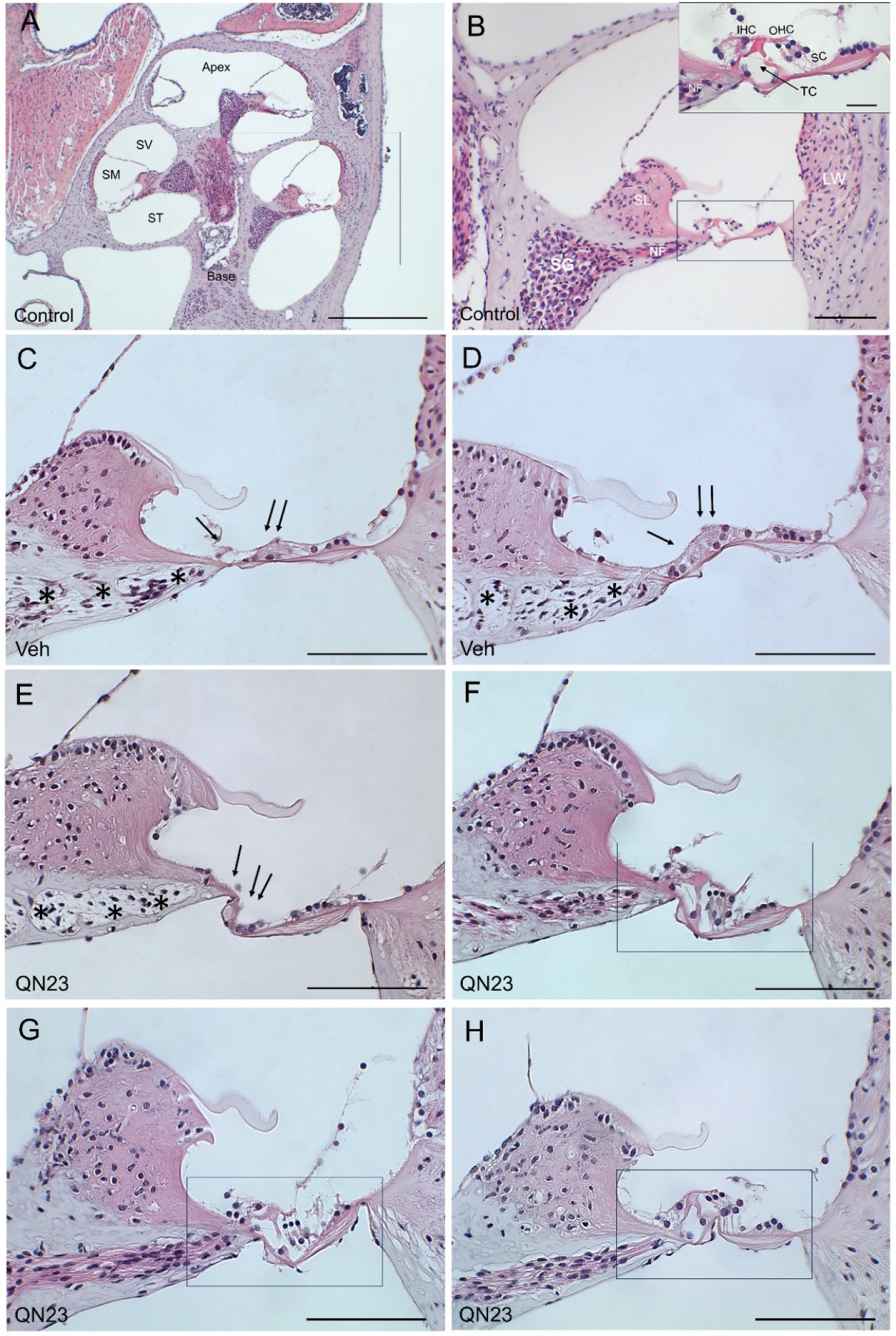
Cochlear cytoarchitecture after noise exposure. Hematoxylin–eosin-stained micrographs showing the morphology of representative cochleae of control mice (*n* = 4); mice injected with vehicle (*n* = 4) or treated with QN23 (*n* = 4) 28 days after noise exposure. (A) Midmodiolar section in control mice showing cochlear turns, divided into vestibular (SV), tympanic (ST), and middle scale (SM). (B) Detail of the middle scale showing the spiral ganglion (SG), spiral limbus (SL), cochlear nerve fibers (NF) and lateral wall (LW), and magnification (inset) of the auditory receptor (organ of Corti) with inner (IHC) and outer (OHC) hair cells, supporting cells (SC) and the tunnel of Corti (TC). (C, D) Organ of Corti of vehicle-injected mouse after noise exposure, showing severe cytoarchitectural changes such as loss of cellular elements (arrows) and nerve fibers (asterisks) at the basal (C) and extended to the basal-middle (D) cochlear regions. (E, F) Organ of Corti from a mouse treated with QN23, showing an altered cytoarchitecture at the basal region (E) but preserved structures at the basal-middle region (F). (G, H) Organ of Corti at the basal cochlear turn in two animals treated with QN23, showing normal cytoarchitecture throughout the cochlea with no apparent changes in the cytoarchitecture. Scale bar: 500 μm (A); 100 μm (B–H); 25 μm (inset B).

### Effect of QN23 on Noise-Induced Alterations in Stress Oxidative and Inflammatory Response Gene Transcription

To investigate if QN23 modifies the impact of noise on the expression of genes involved in oxidative stress and inflammation, cochlear samples were collected, and the expression of key genes was measured by RT-qPCR. First, we evaluated whether the administration of QN23 (three injections, 2 mg/kg every 12 h) itself induced any alterations in cochlear gene expression compared to untreated animals. The results indicated that QN23 did not cause significant changes in the expression levels of *Nrf2* transcription factor and its target genes *Hmox1* and *Nqo1*, encoding detoxication enzymes. Similarly, the expression of the *Nlrp3* inflammasome component and inflammatory cytokines *Tnfa*, *Il1b*, *Il6*, *Il10*, and *Tgfb* was not modified after QN23 treatment (Figure 4).

**Figure 4:**
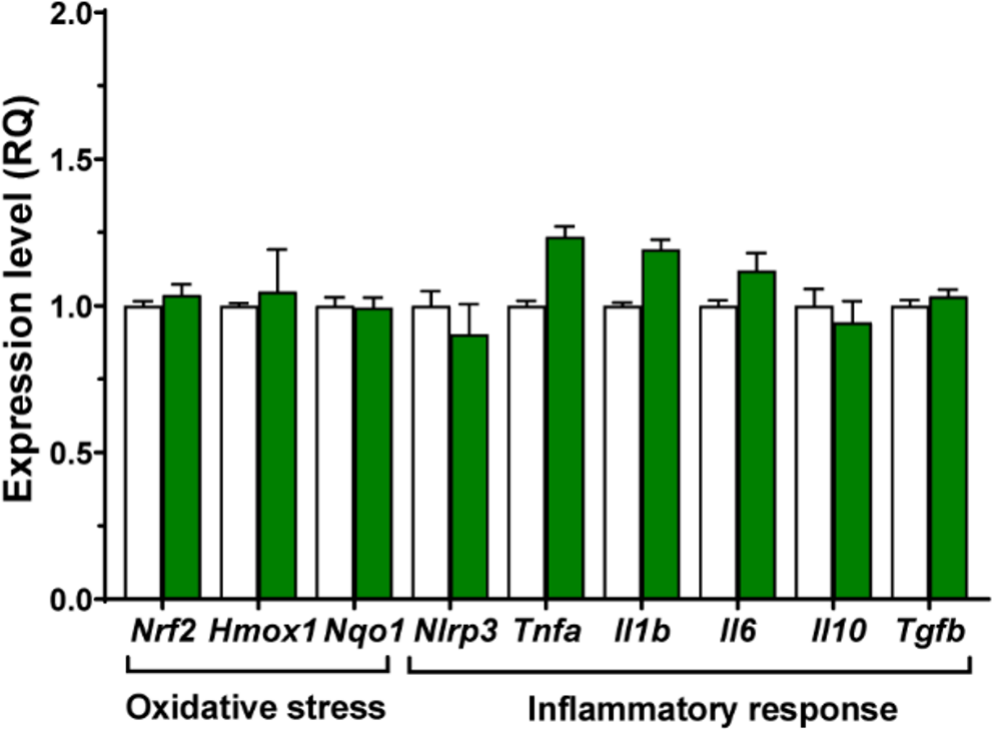
Systemic QN23 administration does not modify cochlear gene expression. Three injections of QN23 at a dose of 2 mg/kg every 12 h were administered before cochlear sampling. RT-qPCR was used to measure expression levels of genes related to oxidative stress and inflammation in pooled cochlear samples from three mice per condition. The expression levels in QN23-injected mice (green bars) were calculated as 2^–ΔΔCt^ (RQ) from three independent experiments performed in triplicate, using *Rplp0* as the reference gene and normalized with respect to control mice (white bars). All data are presented as mean ± SEM. Statistical significance between groups was determined using multiple *t* tests with Holm–Sidak correction.

Next, we evaluated the effect of noise on the transcription of cochlear oxidative stress, inflammatory response, and apoptosis-related genes, 45 min and 24 h after exposure, in the absence or presence of QN23 (Figure 5). We observed a significant increase in *Nrf2* expression after noise exposure (1.5- and 1.3-fold at 45 min and 24 h after injury, respectively) and, accordingly, in the expression of the NRF2 target gene *Hmox1* (10- and 4-fold at 45 min and 24 h, respectively). Noise had been reported to alter the cochlear expression of *Nrf2* and targeted genes.^19,47^ Concretely, the upregulation of *Hmox1*, coding for the HO-1 protein, has been described in the molecular response to noise in mice, rats, and guinea pigs.^36,47–49^ Our results confirmed the noise-induced activation of the cochlear response to oxidative stress by activating the NRF2/HO-1 pathway. *Nox4* expression was not modified by noise exposure at the times studied.

**Figure 5:**
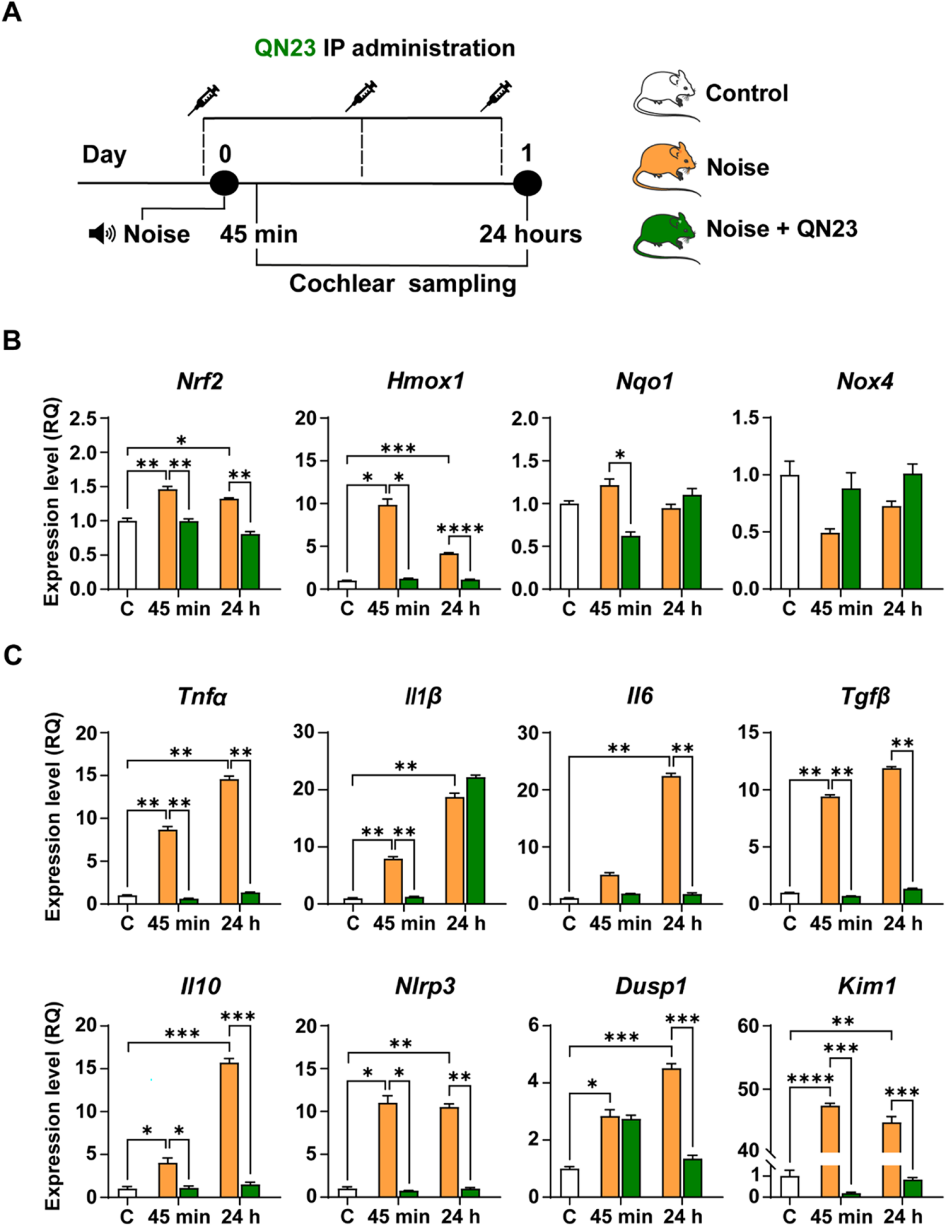
QN23 protection against noise-induced changes in the expression of genes related to the response to oxidative stress. (A) Schematic overview of the experimental design. Briefly, 2-month-old male C57BL/6JCrl mice were exposed to noise for 30 min to induce NIHL either untreated or treated with QN23 at a dose of 2 mg/kg every 12 h. Treatment began 1 h before noise exposure, and cochlear samples were collected at 45 min and 24 h postexposure. RT-qPCR was used to measure the expression levels of genes related to oxidation in pooled cochlear samples from three mice per condition. (B, C) Expression of genes related to oxidate stress (B) and inflammation (C) in mice nonexposed (white bars) or mice exposed to the noise either untreated (orange bars) or treated with QN23 (green bars) was studied 45 min and 24 h postnoise exposure. Gene expression is shown as 2^–ΔΔCt^ (RQ) method from triplicate measurements, with *Hprt1* serving as the reference gene and normalized to the levels observed in control mice (white bars). Data are presented as mean ± SEM. Statistical significance was determined by Brown–Forsythe and Welch ANOVA with T2 multiple comparison tests (only statistically significant data were marked; symbols represent *p*-values of <0.05 (*), < 0.01­(**), < 0.001 (***), and <0.0001 (****), respectively).

In this context, the administration of QN23 reverted the effect of noise on oxidative stress-related genes, with QN23-treated mice showing a gene expression profile similar to that of nonexposed mice (Figure 5). Thus, the expression of genes upregulated by noise, *Nrf2* and *Hmox1*, was normalized by the QN23 treatment, confirming the otoprotective action of the nitrones. The NRF2/HO-1 pathway had been reported to be modulated by TBN, a tetramethylpyrazine nitrone, in a mouse model of amyotrophic lateral sclerosis.^50^

Excessive ROS levels after noise exposure activate the NF-κB and MAPK signaling cascades in the cochlea, leading to the upregulation of immune-related genes and subsequent production of proinflammatory cytokines (TNF-α, IL-1β, IL-6, and TFG-β), as well as chemokines and adhesion molecules, demonstrating the interplay between oxidative stress and inflammation.^21,22,46,51^ ROS are key factors in the stimulation of the inflammasome; thus, activation of NLRP3 and, accordingly, production of IL-1β and IL-18 have been reported following excessive noise exposure^52^

Thus, the expression levels of *Nlrp3*, *Tgfb*, *Tnfa*, *Il1b*, *Il6*, and *Il10* were measured 45 min and 24 h following the noise challenge (Figure 5). Noise also induced a significant increase in the cochlear expression of *Dusp1* and *Kim1*. Dual-specificity phosphatase 1 (DUSP1) is a key regulator of stress-activated protein kinases and *Dusp1* knockout mice show a premature and accelerated SNHL associated with cochlear inflammation, redox imbalance, hair cell death and increased susceptibility to NIHL,^53^ suggesting a critical role in controlling the cross-talk between oxidative stress and inflammation.^54^ KIM-1 is a transmembrane glycoprotein that is upregulated during the apoptotic response,^55,56^ whose expression is upregulated in aged *Dusp1* KO mice but not in those treated with NAC.^54^ Here we show an ≈50-fold increase of *Kim1* cochlear expression in noise-exposed mice, being up to our knowledge the first report of *Kim1* as a potential marker of NIHL (Figure 5). QN23 treatment counteracted noise-induced expression changes in *Dusp1* and *Kim1*.

Altogether, these results confirm that oxidative stress and inflammatory responses are strongly activated early after noise exposure and that, by mitigating these early responses, nitrones prevent acute NIHL. Hearing loss secondary to chronic noise exposure, as well as age-related hearing loss, is also linked to an accumulation of oxidative damage in cochlear cells and could be potentially prevented by QN23. Furthermore, we have previously shown that long-term NAC administration reduces oxidative stress and inflammation and protects hearing in a genetic model of early-onset age-related hearing loss.^53,54^ The challenge with QN23 lies in optimizing dosage and delivery methods to ensure long-term therapeutic levels in cochlear tissues.

In conclusion, the *in vitro* and *in vivo* findings presented here demonstrate that early intervention with QN23, used as a single therapeutic agent, enhances the survival of HEI-OC1 hair-cell-like structures following H_2_O_2_-induced damage and facilitates the recovery of auditory function in noise-exposed mice. These results highlight QN23′s ability to mitigate oxidative stress in cochlear cells while preventing the activation of pathological processes, such as inflammation, that could further compromise cochlear cytoarchitecture and lead to functional impairments.

## Methods

### Nitrones Synthesis

Quinolylnitrone QN23 ((*Z*)-*N*-*tert*-butyl-1-(2-chloro-6-methoxyquinolin-3-yl)­methanimine oxide) and Cholesterol nitrone F2 were synthesized as described in refs 32,39 . Chemical synthesis of nitrones TN1–TN8 is described in ref 40.

### HEI-OC1 Cell Line Culture, Cell Viability, and Nitrone Preparation

Nitrones were tested in HEI-OC1 mouse auditory cells derived from the Immortomouse postnatal organ of Corti, following protocols published by the group.^13,57^ HEI-OC1 cells were cultured in DMEM medium supplemented with 10% fetal bovine serum (Gibco-Thermo Fisher Scientific, Waltham, MA) and 2.5 ug/mL Amphotericin B (A2942, Sigma-Aldrich, Saint Louis, MO). Nitrones QN23, F2, and TNs were dissolved in absolute EtOH ≥ 99.5% (#107017; Merck, Kenilworth, NJ) at 20, 5, or 10 mM (stock solution), respectively, and sonicated for 10 min at 37 °C in a Branson Bransonic M Mechanical Bath (Emerson Electric). Then, they were tested first for ototoxicity at different concentrations in DMEM without FBS, in the absence or presence of H_2_O_2_ (range 1–1.4 mM), and subsequently their efficacy.

*N*-acetylcysteine (NAC) (0.5 mM; Sigma-Aldrich, Saint Louis, MO) was dissolved in 0.01 M PBS (pH 7.4) and used as a reference antioxidant.

Cell viability was evaluated by the XTT method by following the manufacturer′s instructions (Cell Proliferation Kit II, Roche Molecular Systems). Absorbance measurements at 450 and 660 nm were taken with the VERSAmax plate reader (Molecular Devices, San Jose, CA) at 4, 6, and 23 h after the addition of XTT. All reading times rendered similar results. Cell viability was calculated as follows: Specific absorbance = [Abs_450_ nm (test) – Abs_450_ nm (mock)] – Abs_660_ nm (test). Control wells without seeded cells were used as a reference.

### Nitrone Preparation for *In Vivo* Administration

The described experimental activities have undergone local institutional review assessing safety and humane usage of study subject animals. Experimental procedures with mice followed European Community 2010/63/EU and Spanish RD 53/2013 guidelines and were reviewed and approved by the local and institutional Ethics committee and finally authorized by the regional competent authority (PROEX 281.3/20 and 325.4/21). The experiments were carried out in C57BL/6JCrl mice (Charles River Laboratories, Barcelona, Spain), male, aged 1.5 months at the beginning of the study. Three independent experiments were performed, with *n* = 6–10 mice per group. QN23 was prepared as described in ref 39. Briefly, QN23 was dissolved in ethanol, poly­(ethylene glycol) (PEG400, Sigma), and saline (1:200:60, by vol) to obtain a stock solution-1 (3.6 mg/mL). The stock solution-1 was then diluted in poly­(ethylene glycol) and saline (6:7, by volume) to obtain the stock solution-2, 0.25 mg/mL (854 μM). QN23 was prepared freshly before each administration and injected intraperitoneally in 2 h following its preparation. QN23 was administered at 2 mg/kg (8 mL/kg) twice a day for 3 days, with the first dose injected 1 h before noise exposure. The vehicle solution was prepared identically, and control animals received a similar volume (8 mL/kg).

### Auditory Evaluation and Noise Exposure

Hearing was assessed by recording auditory brainstem evoked potentials (ABR) before (baseline) and 1, 14, and 28 days after noise exposure, as described.^48^ Briefly, click and tone-burst stimuli (4, 8, 16, 24, and 32 kHz) were presented with an MF1 magnetic speaker (TDT) from 90 to 20 dB SPL in 5–10 dB SPL steps. Click stimuli were 0.1 ms, and tone-burst stimuli were 5 ms in duration (2.5 ms each for rise and decay, without plateau). The thresholds of click-evoked and tone-evoked ABRs, peak latencies, and amplitudes were determined. Mice were exposed to 2–20 kHz, 105 dB SPL noise for 30 min as described.^4,46^

### Cochlear Morphology and Gene Expression

Inner ear samples were collected 28 days after noise exposure for histological study. Mice were euthanized by pentobarbital overdose and intracardially perfused with PBS 0.1 M and then PFA 4% in PBS 0.1 M. Cochleae were dissected and postfixed in PFA 4% overnight, washed in PBS 0.1 M, decalcified with EDTA 5% for 7 days, and embedded in paraffin wax. Paraffin sections (7 μm) were stained with hematoxylin–eosin (Sigma) to evaluate cochlear cytoarchitecture.

For gene expression studies, cochleae from nonperfused animals were dissected and immediately frozen. Cochlear RNA was extracted with an RNeasy kit (QIAGEN, Hilden, Germany). Quality and quantity of RNA were determined with an Agilent Bioanalyzer 2100 instrument (Agilent Technologies, Santa Clara, CA). RNA from three animals per experimental group was pooled. cDNA was then generated by reverse transcription (High-Capacity cDNA Reverse Transcription Kit; Applied Biosystems, Foster City, CA), and gene expression was analyzed in triplicate by qPCR on Applied Biosystems 7900 HT using TaqMan Gene Expression Assays (ThermoFisher Scientific) for *Nrf2*, *Hmox1*, *Nqo1*, *Nox4*, *Nlrp3*, *Tnfa*, *Tgfb*, *Il1b*, *Il6*, *Il10*, *Kim1*, and *Dusp1*. *Rplp0* or *Hprt1* was used as an endogenous housekeeping control gene (Supporting Table 1). Gene expression was calculated as 2^–ΔΔCt^ (RQ).

## Supplementary Material



## Notes

TaqMan probes used for RT-qPCR assays (PDF)

## Abbreviations

ABR: auditory brainstem responses
NIHL: noise-induced hearing loss
QN: quinolylnitrone
NAC: *N*-acetyl-l-cysteine
ROS: reactive oxygen species
RNS: reactive nitrogen species
PBN: phenyl-*N*-*tert*-butylnitrone
NXY-059: disufenton sodium
4-OHPNB: 4-hydroxy PBN
TN: triazole-derived nitrone
