# Supplementary material for: Quinolylnitrone 23 Protects from Auditory Cell Oxidative Injury and Noise-Induced Hearing Loss
Source: ACS Pharmacol Transl Sci. 2025 Aug 26;8(9):3007–18. doi: 10.1021/acsptsci.5c00221 (PMC12441856; doi:10.1021/acsptsci.5c00221)
Supplement: Supplementary file 1 [file pt5c00221_si_001.pdf]

SUPPORTING INFORMATION

Quinolyl nitrone 23 protects from auditory cell oxidative injury and noise-induced hearing loss

Silvia Murillo-Cuesta[1,2,3,], Julio Contreras[1,2,4], Mourad Chioua[5], Carmen García-Montoya[1,2], Lourdes Rodríguez-de la Rosa[1,2,3], Inés Méndez-Grande[1,2], Dorota G. Piotrowska[6], Iwona E. Głowacka[6], Isabel Varela-Nieto[1,2,3], José Marco-Contelles[2,5]*

1 Institute for Biomedical Research "Sols-Morreale", Spanish National Research Council-Autonomous University of Madrid (CSIC-UAM), 28029 Madrid, Spain.

2 Centre for Biomedical Network Research on Rare Diseases (CIBERER), Institute of Health Carlos III (ISCIII), 28029 Madrid, Spain.

3 Hospital La Paz Institute for Health Research (IdiPAZ), 28046 Madrid, Spain.

4 Anatomy and Embryology Department, Faculty of Veterinary, Universidad Complutense de Madrid, 28040 Madrid, Spain

5 Laboratory of Medicinal Chemistry, Institute of Organic Chemistry (CSIC), Madrid 29006, Spain.

6 Bioorganic Chemistry Laboratory, Faculty of Pharmacy, Medical University of Lodz, Muszynskiego 1, 90-151 Lodz, Poland.

[*]Corresponding authors: Silvia Murillo-Cuesta (silvia.murillo@csic.es), Isabel Varela-Nieto (i.varela.nieto@csic.es)

Table of contents

Supplementary Table S1……………………………………………………………page S-2

Supplementary Table 1. TaqMan probes used for RT-qPCR assays.

Gene symbol	Gene name	Reference
**Oxidative stress response-related genes**		
NFE2L2 (Nfr2)	*Nuclear factor, erythroid derived 2, like 2*	Mm00477784_m1
Hmox1 (Ho1)	*Heme oxygenase 1*	Mm00516005_m1
Nqo1	*NAD(P)H dehydrogenase, quinone 1*	Mm01253561_m1
Nox4	*NADPH oxidase 4*	Mm00479246_m1
**Inflammation-related genes**		
Nlrp3	*NLR family, pyrin domain containing 3*	Mm00840904_m1
Tnfa	*Tumor necrosis factor alpha*	Mm99999068_m1
Tgfβ1	*Transforming growth factor, beta 1*	Mm01178820_m1
Il1b	*Interleukin 1 beta*	Mm00434228_m1
Il6	*Interleukin 6*	Mm00446190_m1
Il10	*Interleukin 10*	Mm00439614_m1
Dusp1	*Dual specificity phosphatase 1*	Mm00457274_g1
Havcr1 (Kim1)	*Hepatitis A virus cellular receptor 1*	Mm00506686_m1
**Endogenous reference genes**		
Rplp0	*Ribosomal protein, large, P0*	Mm00725448_s1
Hprt1	*Hypoxanthine Phosphoribosyltransferase 1*	Mm05914375_s1
